# Influence of hydrofluoric acid treatment on electroless deposition of Au clusters

**DOI:** 10.3762/bjnano.8.19

**Published:** 2017-01-18

**Authors:** Rachela G Milazzo, Antonio M Mio, Giuseppe D’Arrigo, Emanuele Smecca, Alessandra Alberti, Gabriele Fisichella, Filippo Giannazzo, Corrado Spinella, Emanuele Rimini

**Affiliations:** 1 CNR-IMM Institute for the Microelectronics and Microsystems, Z. I. VIII Strada 4, Catania, I-95121, Italy; 2 Department of Physics and Astronomy, v. S. Sofia 64, I-95123, Catania, Italy

**Keywords:** electroless deposition, galvanic deposition, gold nanoparticles, HF acid treatment, HF-propelled motion, hydrogen termination, silicon surfaces

## Abstract

The morphology of gold nanoparticles (AuNPs) deposited on a (100) silicon wafer by simple immersion in a solution containing a metal salt and hydrofluoric acid (HF) is altered by HF treatment both before and after deposition. The gold clusters are characterized by the presence of flat regions and quasispherical particles consistent with the layer-by-layer or island growth modes, respectively. The cleaning procedure, including HF immersion prior to deposition, affects the predominantly occurring gold structures. Flat regions, which are of a few tens of nanometers long, are present after immersion for 10 s. The three-dimensional (3D) clusters are formed after a cleaning procedure of 4 min, which results in a large amount of spherical particles with a diameter of ≈15 nm and in a small percentage of residual square layers of a few nanometers in length. The samples were also treated with HF after the deposition and we found out a general thickening of flat regions, as revealed by TEM and AFM analysis. This result is in contrast to the coalescence observed in similar experiments performed with Ag. It is suggested that the HF dissolves the silicon oxide layer formed on top of the thin flat clusters and promotes the partial atomic rearrangement of the layered gold atoms, driven by a reduction of the surface energy. The X-ray diffraction investigation indicated changes in the crystalline orientation of the flat regions, which partially lose their initially heteroepitaxial relationship with the substrate. A postdeposition HF treatment for almost 70 s has nearly the same effect of long duration, high temperature annealing. The process presented herein could be beneficial to change the spectral response of nanoparticle arrays and to improve the conversion efficiency of hybrid photovoltaic devices.

## Introduction

Gold nanoparticles on silicon substrates have shown quite interesting applications in the fields of Si nanowire (SiNW) catalysis [1–3], metal-assisted etching (MAE) [4] or even as electrical contacts in standard miniaturized devices [5]. Their ability to display enhanced surface plasmon resonance (SPR) at optical frequencies makes them excellent at scattering and absorbing visible light [6–8]. For these reasons, they have found an interesting application in the field of metal–semiconductor hybrid structures for solar energy conversion [9–10]. Among the different adopted methods to deposit Au nanoclusters on a substrate, electroless deposition based on galvanic displacement reactions is an efficient and versatile technique. It consists of manually dipping samples in a plating bath for few seconds without the need for application of an external current or potential [11–15]. Details about the reaction between Au^+^ and Si atoms involved in the deposition are described elsewhere [16]. The morphology of gold by electroless deposition is quite complex and a number of basic questions remain to be clarified, such as the nature of the interface with silicon in the presence of hydrofluoric acid (HF). It has been reported in some studies that silicides are formed due to the strong interaction of Au atoms with Si [17]. On this topic, several contrasting reports are found in the literature but it is generally accepted that at a critical thickness (in the range of 2 to 5 ML) the gold seems inert and unable to mix with silicon. For gold deposited on silicon via galvanic displacement (GD), both Volmer–Weber and Stranski–Krastanov modes of growth have been suggested to be involved. It has been found that Si atoms diffuse outwards through the deposited gold layers during the growth process with the subsequent formation of Si oxide on their surface. The process stops after a certain thickness of oxide is formed and on top of it gold atoms agglomerate as solid clusters [18–21]. The optical properties of these gold clusters depend on their shape and morphology. It is reported in literature that the local field enhancement factor of gold nanoparticles depends on their geometry and it is higher for well-shaped spherical particles than for flat islands [22]. Generally, an additional postdeposition annealing step is usually required to improve the spectral response. In our previous study on the GD of Au^+^ ions onto silicon substrates, we found that metal nanoparticles nucleate instantaneously and their subsequent growth is governed by diffusion in the solution [23]. In detail, we showed that by immersion of a Si(100) substrate for a few seconds in a solution containing 1 mM KAuCl_4_ and 4.8 M HF, AuNPs with a mean radius of less than 10 nm and a density higher than 10^10^ cm^−2^ are formed. In the present work, we show that with proper HF treatments, the 3D Au clusters prevail over the flat regions obtained by layer-by-layer growth. Such a beneficial alteration requires only a manual immersion for a few seconds in a diluted hydrofluoric acid solution (DHF) with 6% HF without the need for annealing or high power light irradiation [24].

## Results and Discussion

**Figure 1:**
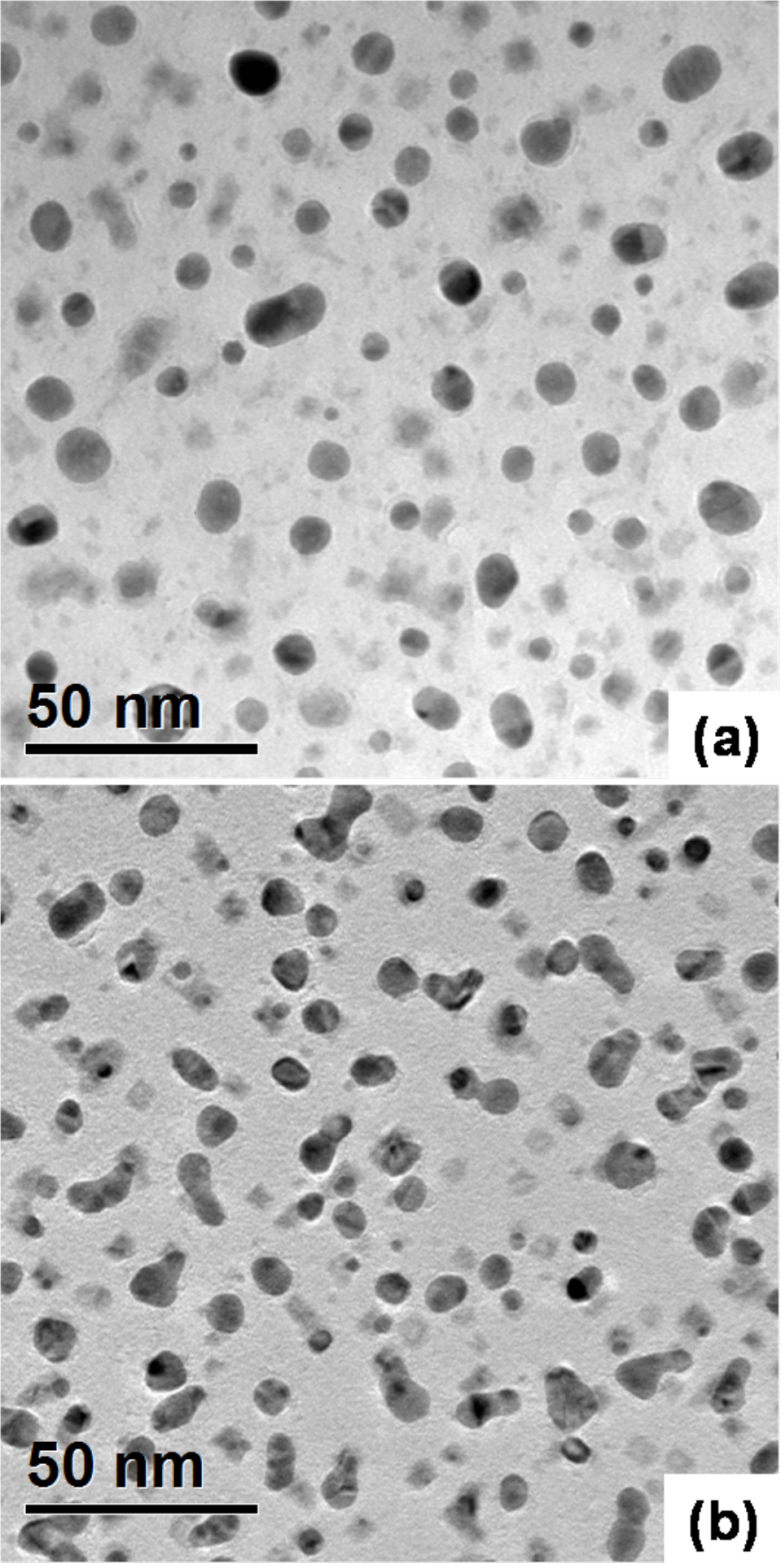
Plan view TEM micrographs of AuNPs electroless deposited on a Si substrate by immersion for 3 s in the solution after a DHF pretreatment of 240 s; sample preparation (a) standard high energy and (b) gentle milling procedure.

The morphology of the samples was investigated with transmission electron microscopy (TEM). It has been reported in previous papers that the specimen preparation could alter the original and structural characteristics of the Au particles [25]. Therefore, we prepared the samples for TEM analysis using two different thinning procedures. One sample was obtained after a conventional TEM preparation technique, including mechanical and high energy ion thinning (Ar^+^ ions at 5 keV). The other one was prepared instead with the so-called “gentle milling” procedure using low energy (0.1–1 keV) Ar^+^ ions (see details in the Experimental section). The results are compared in Figure 1 that shows a plan view TEM of the silicon substrate after the same gold deposition process but prepared following the standard (Figure 1a) and the gentle milling (Figure 1b) procedures, respectively. The morphology is drastically modified by the high energy Ar^+^ ion thinning, where the mean particle size is about 20% larger and the density increases by about 30% with respect to the low energy Ar^+^ beam thinning procedure. The particles in Figure 1a are more spherical in shape with light grey areas of irregular shape, while in Figure 1b, the clusters are elongated and we observe square light gray regions. Clearly, gold atom rearrangement occurs in the sample during the standard thinning process that probably results from the heating of the sample due to the higher Ar^+^ ion energy. In order to avoid artifacts, we therefore adopted the gentle milling procedure for all the samples to be analyzed by TEM. We tested the reliability of the mechanical thinning procedure by repeating both the HF pretreatments and the deposition step on a Si sample already thinned and then ready for TEM analysis. The Au cluster morphology was quite similar (see Supporting Information File 1, Figure S1) to that obtained by deposition on bulk substrates. As a result, we used standard wafers for deposition, and the samples were mechanically thinned and gently milled for TEM observations.

### HF treatment before Au deposition

**Figure 2:**
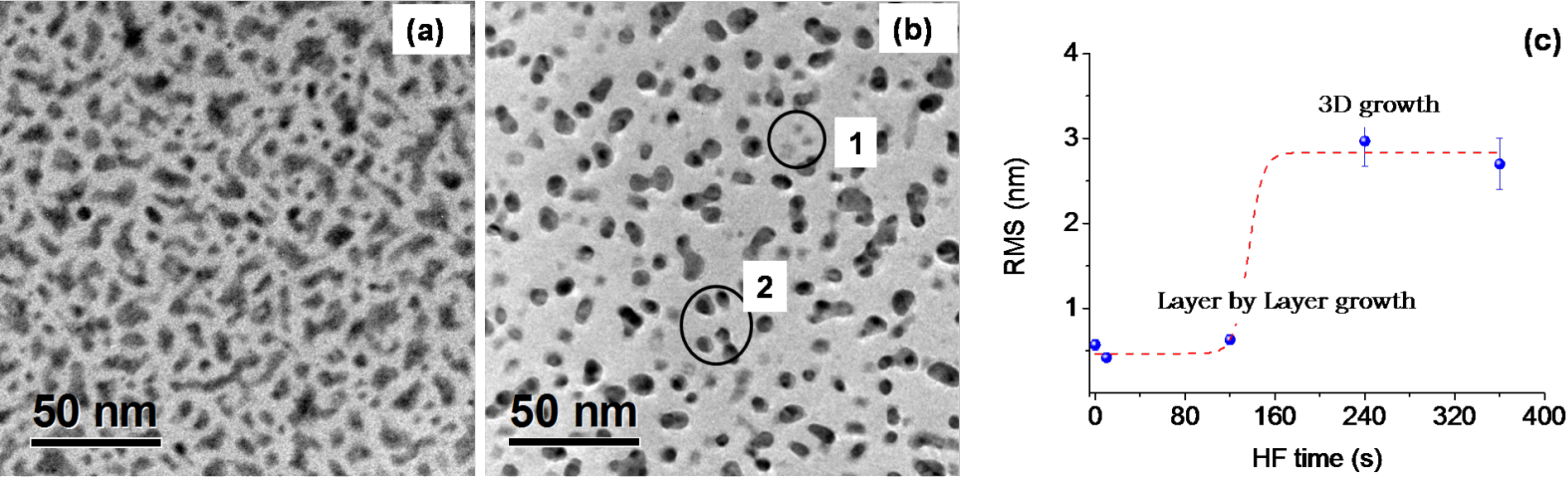
Gold electroless deposition on Si(100) after an DHF pretreatment of 10 s (a) and 240 s (b); root mean squared (RMS) for the corresponding Si substrates (c) measured with AFM.

The Si(100) substrates were cleaned following the procedure described in the experimental section. Some of them were subsequently immersed in DHF (diluted HF, 6% HF) for 10 s and some others for 240 s. The electroless gold deposition takes place then by immersion in the plating solution for 3 s. The TEM images of the corresponding samples are reported in Figure 2a,b, respectively. The gold morphology clearly varies with the time of the DHF pretreatment. After 10 s in DHF (see Figure 2a), Au atoms uniformly assemble on the Si substrate with elongated and wide structures of a few tens of nanometers long. After 4 min in DHF (Figure 2b), they arrange as small particles of spherical shape with a radius of less than 10 nm. Although the images were taken in bright field mode, a huge mass contrast between light gray, thin regions of square shape (labeled 1 in Figure 2b) and the dark, thick round particles (labeled 2 in Figure 2b) is clearly shown.

The two samples were also analyzed by Rutherford backscattering (RBS) measurements with 2 MeV He^+^ ions in order to obtain the areal density of the deposited gold atoms, which was found to be 4.24 × 10^15^ atoms cm^−2^ for sample (a) and to 4.73 × 10^15^ atoms cm^−2^ for sample (b). By comparing these results with the fractional covered area measured by the TEM micrographs we estimated an equivalent mean thickness of 3 nm and 10 nm, respectively. So, in other words, in the first case the layer-by-layer growth prevails while a 3D arrangement is promoted by the DHF pretreatment of 240 s. It is well known that HF strongly modifies the silicon surface roughness [26–29] and wetting properties [30]. The surface-free energy of gold is 1410 × 10^−3^ J/m^−2^, while that of Si and SiO_2_ are 1240 × 10^−3^ J/m^2^ and 400 × 10^−3^ J/m^2^, respectively [31]. The native SiO_2_ is completely etched after 10 s (etch rate 500 Å/min) so nucleation occurs on the hydrogenated silicon surface [32]. The roughness of the Si surface after a pretreatment of 4 min in DHF was measured by AFM, and the obtained root mean square (RMS) was 3 nm, which is about one order of magnitude higher than a typical Si wafer (Figure 2c). Consequently, the surface-free energy increases [33] for a rougher surface and promotes 3D cluster formation by offering preferential sites for nucleation as apex or surface discontinuities.

### HF postdeposition treatment

**Figure 3:**
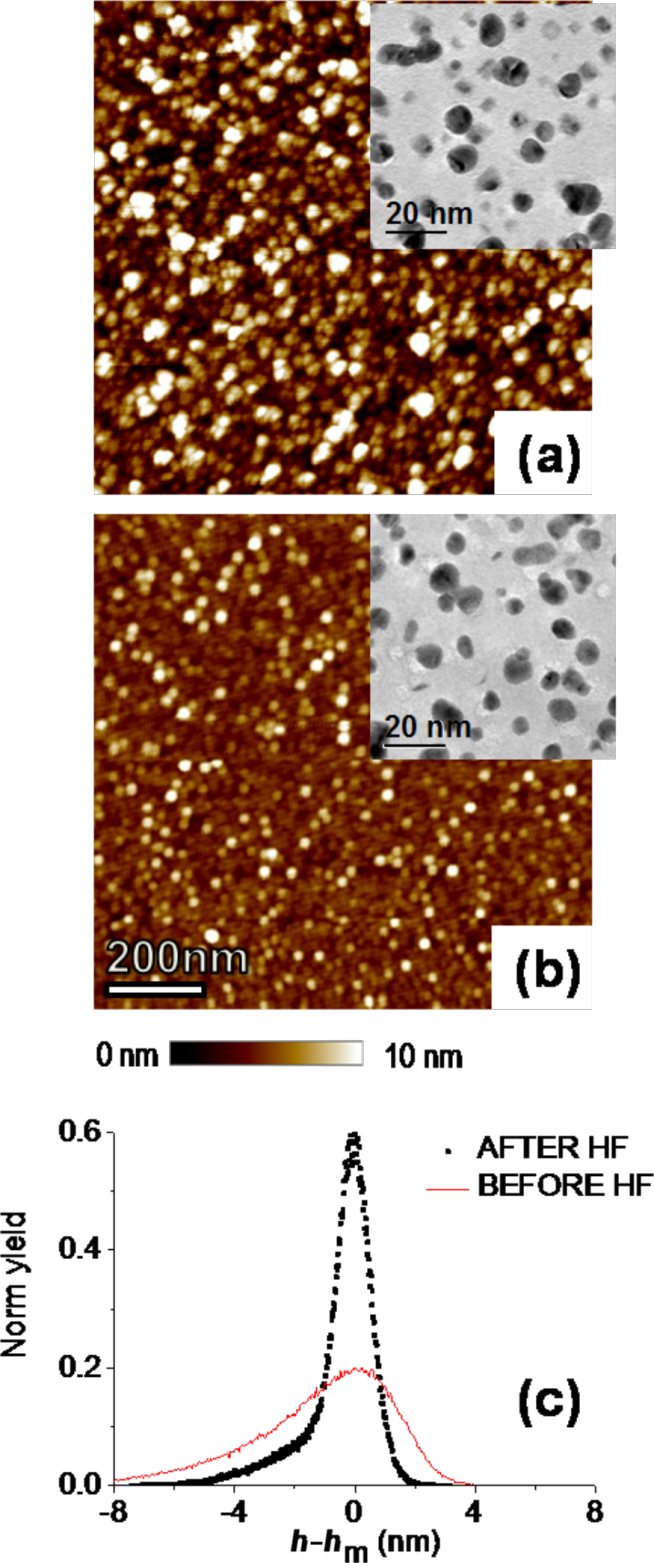
AFM *z*-scan of Si sample with AuNPs before (a) and after (b) a postdeposition bake in HF for 70 s, with the corresponding TEM images in the upper right hand part. (c) Height distribution relative to the mean value, as calculated from AFM images.

So far we have shown the influence of a DHF pretreatment on the subsequent Au deposition regarding the shape and morphology of the nanoclusters. We now consider what happens if the DHF treatment is performed after the gold deposition. There are many papers dealing with dynamic coalescence of metal nanoparticles in liquids [34–36]. In a previous work, we found that silver nanoparticles are subjected to Smoluchowski [37] ripening in DHF solutions by increasing their size and decreasing their surface density. For the case of gold nanoparticles on Si, we expect a more complex scenario due to the stronger interaction between gold and silicon with the formation of quasi-heteroepitaxial layers. Figure 3 reports the morphologies observed by AFM (in tapping mode with a silicon tip of 10–15 nm curvature radius) of Si samples with AuNPs before (Figure 3a) and after (Figure 3b) an HF postdeposition treatment of 70 s.

The postdeposition treatment clearly results in a much flatter surface morphology than in the untreated sample. This effect can be quantified by calculating the root mean square (RMS) roughness from the two 1 × 1 µm scans in Figure 3, showing a decrease in the RMS value from 2.53 nm for the untreated sample to 1.16 nm after the HF treatment. This indicates a more uniform height distribution of clusters with respect to the substrate after the postdeposition treatment. To better illustrate this aspect, the histograms of the *z* values (in terms of deviation from the mean height) extracted from the two AFM images are also reported in Figure 3c, showing a broad distribution in the as-deposited sample, with an asymmetric tail extending toward lower *z* values, whereas a much narrower distribution is observed after the HF treatment.

The corresponding plan view TEM micrographs of the two samples are also reported in the inserts of Figure 3a and Figure 3b, respectively. The selected area electron diffraction (SAED), shown in Supporting Information File 1, Figure S2, of the AuNPs presents a bright spot in the (200)Au ring along the (400)Si directions indicating a heteroepitaxial arrangement of the deposited Au atoms, as also previously reported [21]. The intensity of this spot is reduced after HF treatment so one could envisage that the flat gray regions are heteroepitaxial with the substrate orientation. The 3D arrangement of the Au atoms after the HF solution treatment indicates that the heteroepitaxial relationship with the substrate is partially lost.

Moreover, the amount of Au on the sample, as measured using RBS, does not change even after an HF treatment of 70 s, indicating that the atomic arrangement is not associated to dissolution of the deposited gold atoms (see Supporting Information File 1, Figure S3).

**Figure 4:**
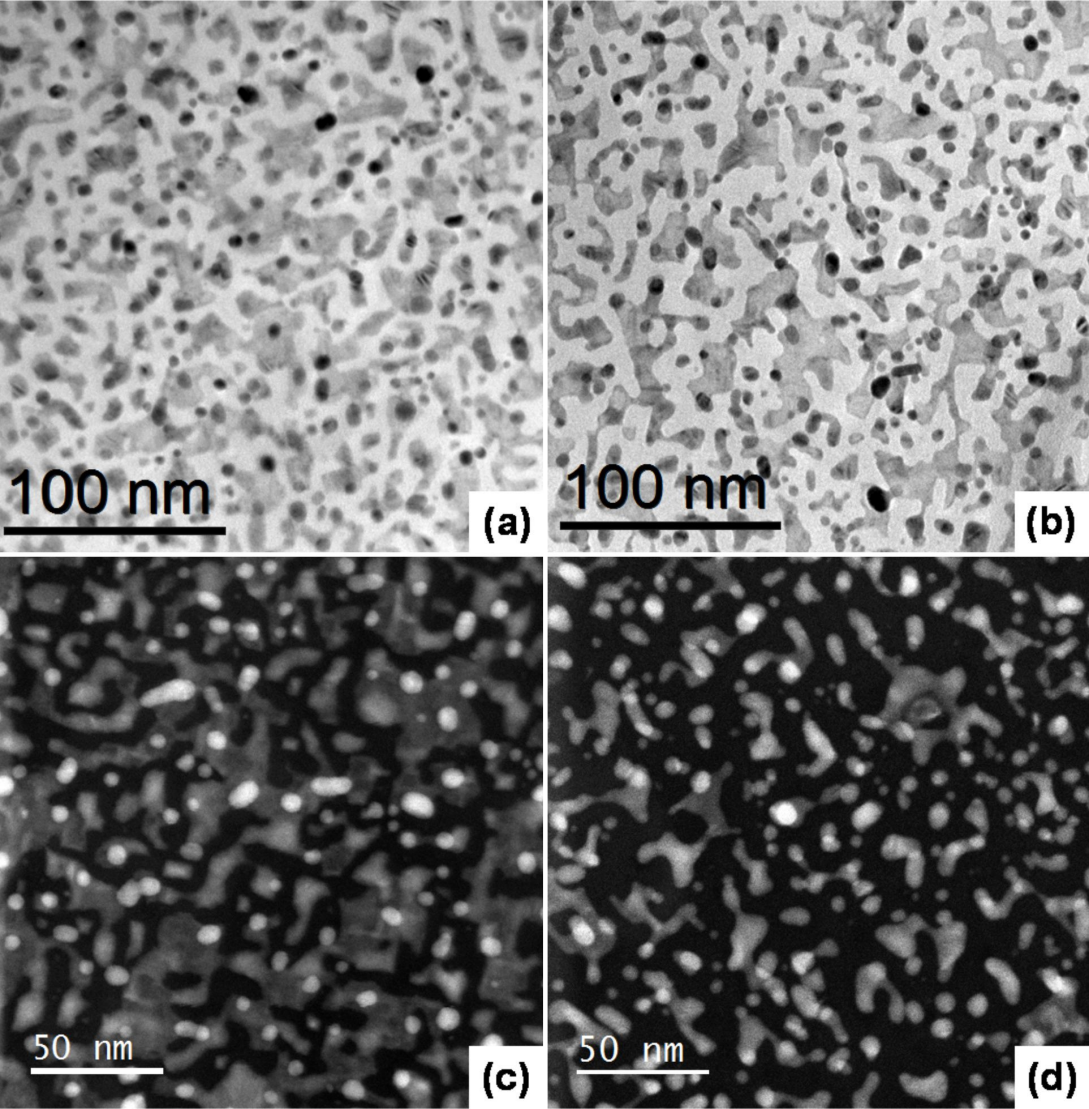
Plan view TEM of Si sample with Au islands obtained with 20 s immersion in the plating solution (a); the same sample after a HF postdeposition treatment of 70 s (b) and (c) and (d) STEM image of (a) and (b), respectively.

For a better understanding of the process taking place under immersion in HF we prepared a sample with large, flat regions, obtained after a short HF pretreatment followed by an immersion for 20 s in the plating solution. The morphology of gold was examined with plan view TEM and scanning transmission electron microscopy (STEM) in high-angle annular dark-field imaging (HAADF) Z-contrast (atomic number) imaging mode, while the crystalline structure was analyzed in detail with X-ray diffraction. Figure 4a and Figure 4b are the plan view TEM of the samples before and after the HF postdeposition treatment for 70 s. The more discernible effect is a pronounced change in the fractional covered area that varies by about 15%. By using a STEM detector with a large inner radius (a HAADF detector) electrons are collected which are not Bragg scattered. As such HAADF images show little or no diffraction effects, and their intensity is approximately proportional to *Z*^2^. The HF postdeposition treatment levels off the intensity distribution of the corresponding STEM micrograph, indicating a more uniform thickness for the gold particles as clearly seen by comparing Figure 4c and Figure 4d, respectively.

**Figure 5:**
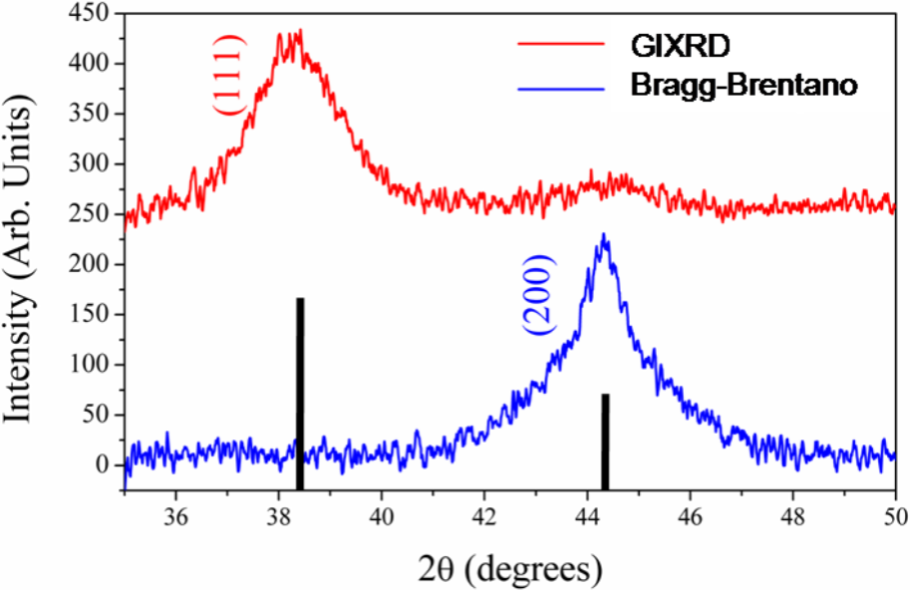
Diffraction profile of sample with AuNPs from a 20 s deposition for the grazing incidence (red, upper) and for the Bragg–Brentano (blue, lower). The bars represent the computed intensities for a random Au powder in the Bragg–Brentano geometry.

The samples were also inspected with X-ray diffraction (Figure 5). Two different configurations were adopted. In the Bragg–Brentano geometry we observed a significant (200) Au peak at 2θ = 44.3°. Although the (111) peak at 38.2° was expected to be the most dominant (the bars represented the computed intensities for a powder sample), the dominant peak at 2θ = 44.3° indicates the occurrence of texturing in the Au cluster orientation. The (111) Au peak at 2θ = 38.2°, measured with grazing incidence X-ray diffraction, showed the presence of randomly oriented Au clusters coexisting with the heteroepitaxial regions on the Si substrate.

**Figure 6:**
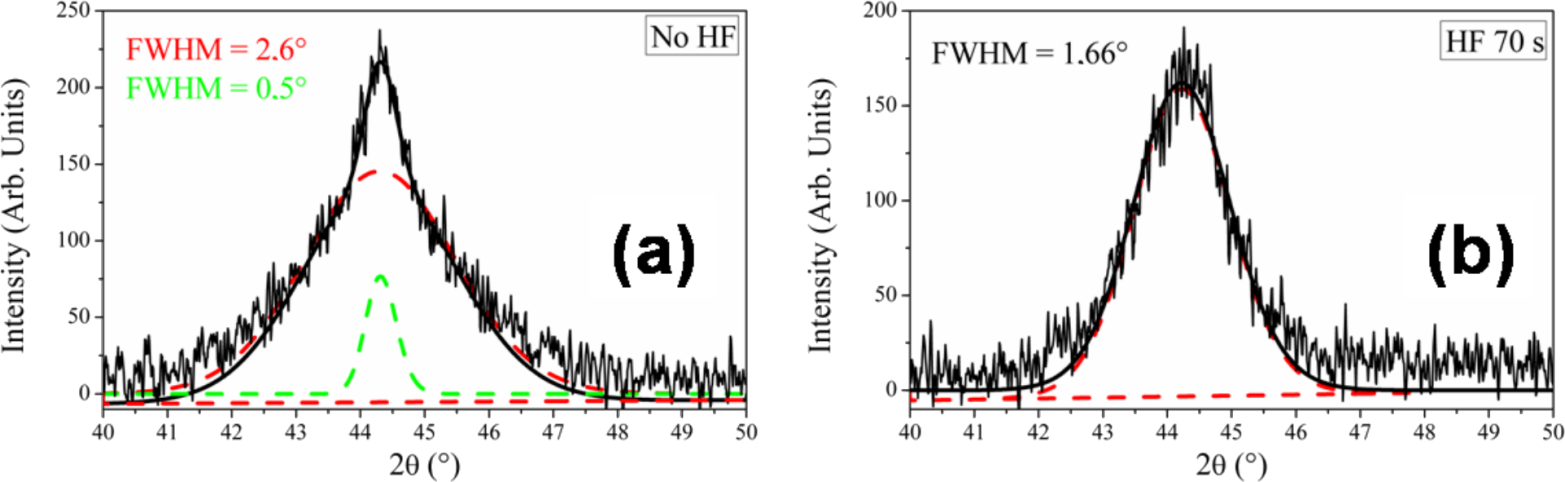
Au (200) peak as measured by XRD in Bragg–Brentano geometry of (a) as-deposited sample and (b) the same sample but after an HF treatment for 70 s.

Moreover, the shape of the (200) diffraction peak indicates that two families of grains, both exhibiting texturing in the [001] crystallographic direction, are present. The deconvolution of the signal is reported in Figure 6a and the size of the corresponding crystals is determined by the Scherrer formula [38].

We observe a broad peak, with a FWHM of 2.6°, associated with large regions about 3 nm thick. They coexist with small 3D clusters that are about 15 nm high and whose contribution to the diffraction profile is represented by the Gaussian shape with a FWHM of 0.5°. The same analysis was repeated after immersion of the sample in HF for 70 s, and the diffractogram is shown in Figure 6b. The FWHM for the XRD profile decreases with increasing time in HF and is 2.26° after 40 s and 1.66° after 70 s, indicating a thickening of the islands along the growth axis (3.8 nm and 5 nm, respectively). The results are in agreement with the TEM images that predicted an “in-plan” shrinkage plus “in-height” increase for gold structures. We probed the modification in the crystalline orientation, as found by SAED, by comparing the “heteroepitaxial ratio”, defined as:

[1]
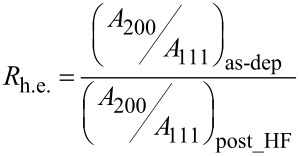


where *A* is the area under the corresponding peak. *R*_h.e._ is a parameter related to the degree of texturing exhibited by the Au particles as a function of the HF treatment. The ratio amounts to 1.17 after 40 s and to 1.75 after 70 s, indicating a reduction of the textured component with immersion time in the HF solution.

## Conclusion

In this work we investigate the morphology of gold aggregates on silicon (100) substrates obtained by galvanic displacement using XRD, AFM and TEM (with a proper low-energy ion milling procedure). It was shown that Au atoms assemble in thin regions and are irregularly shaped and also form in 3D spherical clusters. The 3D spherical clusters form according to the layer-by-layer and island growth modes but it is possible to select between the two by varying the immersion time in DHF solution during the cleaning procedure. Specifically, after a quick immersion time of about 10 s and a deposition time of 3 s, we obtained a discontinuous gold film of a few monolayers thick. When the HF pretreatment is extended up to 4 min, for the same plating time, we observed mostly spherical clusters, while the thin regions were reduced to small squares of a few nanometers long, which were aligned with the [001] direction of the substrate. With a 10 s HF pretreatment and longer deposition times (about 20 s), both structures coexist, corroborating the layer-by-layer plus island growth modes for gold on silicon. The SAED and the XRD measurements showed a pronounced heteroepitaxial component but also a polycrystalline phase for Au on Si(100) obtained by galvanic displacement. The layered gold regions could be altered by means of postdeposition HF treatments. TEM observations and XRD analysis showed that they thicken and rearrange their own crystalline structure.

HF is known to etch SiO_2_, changing the surface chemical composition and also the free energy of Si. As a result, the layered gold is freed and the outlying atoms move toward inner regions to reduce their surface free energy. The process is very fast and bears similarities with the HF self-propelled motion of liquid droplets on solid surfaces [39]. The results are comparable to those obtained after high temperature and/or time consuming annealing procedures.

## Experimental

**Pretreatment of silicon substrate and metal deposition.** The starting substrate was a Si(100) n-type substrate with ρ = 3–5 Ω cm. Prior to plating, each sample was cut into squares of 1.5 × 1.5 cm and degreased in acetone at 60 °C and then placed in an ultrasonic bath for 6 min. This was followed by immersion in DHF (6% HF) for 10 s or 240 s. The substrates were then thoroughly washed with deionized water and blow-dried in air. Gold deposition was carried out by manually soaking each sample in a solution containing 1 mM KAuCl_4_ (99.995% trace metal, Sigma-Aldrich) and 10% HF (diluted HF, GPR RECTAPUR 40%, VWR) at room temperature, under ambient light conditions and without stirring. Then the samples were rinsed in water to remove all surfactants and products. They received an additional cleaning in acetone and were dried in air.

**TEM sample preparation.** For plan view, the samples were mechanically thinned from the backside to less than a micrometer. Then they were milled with a Gatan PIPS II device, according to a low-energy procedure. Gentle milling was performed at low temperature (less than −100 °C with LN_2_ cooling) at a 6° milling angle and with beam energy of 1 keV and current of 30 µA at the beginning and of 0.1 keV and 25 µA for final polishing.

## Supporting Information

We provide images for further understanding of the influence of the TEM thinning procedures and we give evidence of the random rearrangement of gold atoms by electron diffraction on a selected area. We also show the Rutherford backscattering spectra of the samples before and after the HF postdeposition treatments to demonstrate that the amount of gold atoms does not change.

File 1 Additional experimental data.
