# Supplementary material for: Influence of hydrofluoric acid treatment on electroless deposition of Au clusters
Source: Beilstein J Nanotechnol. 2017 Jan 18;8:183–9. doi: 10.3762/bjnano.8.19 (PMC5301911; doi:10.3762/bjnano.8.19)
Supplement: File 1 — Additional experimental data. [file Beilstein_J_Nanotechnol-08-183-s001.pdf]

Supporting Information

for

Influence of HF on electroless deposited Au clusters

Rachela G. Milazzo*[1], Antonio M. Mio[1], Giuseppe D'Arrigo[1], Emanuele Smecca[1], Alessandra Alberti[1], Gabriele Fisichella[1], Filippo Giannazzo[1], Corrado Spinella[1] and Emanuele Rimini[1,2]

Address: [1]CNR-IMM Institute for the Microelectronics and Microsystems, Z. I. VIII Strada 4, Catania, I-95121, Italy and [2]Department of Physics and Astronomy, v. S. Sofia 64, I-95123, Catania, Italy

Email: Rachela Gabriella Milazzo - gabriella.milazzo@imm.cnr.it

* Corresponding author

Additional experimental data

Influence of the thinning TEM techniques

Comparison between AuNPs on Si obtained on bulk samples followed by the gentle milling procedure (Figure S1a) and those obtained by direct deposition on the as thinned silicon sample, but before the Ar+ milling (Figure S1b). Gold morphology did not change within the experimental error in the immersion time (deposition is very fast, about 5×10^{14} atoms cm^{-2} s^{-1}) so we opted for bulk deposition plus gentle milling procedure.

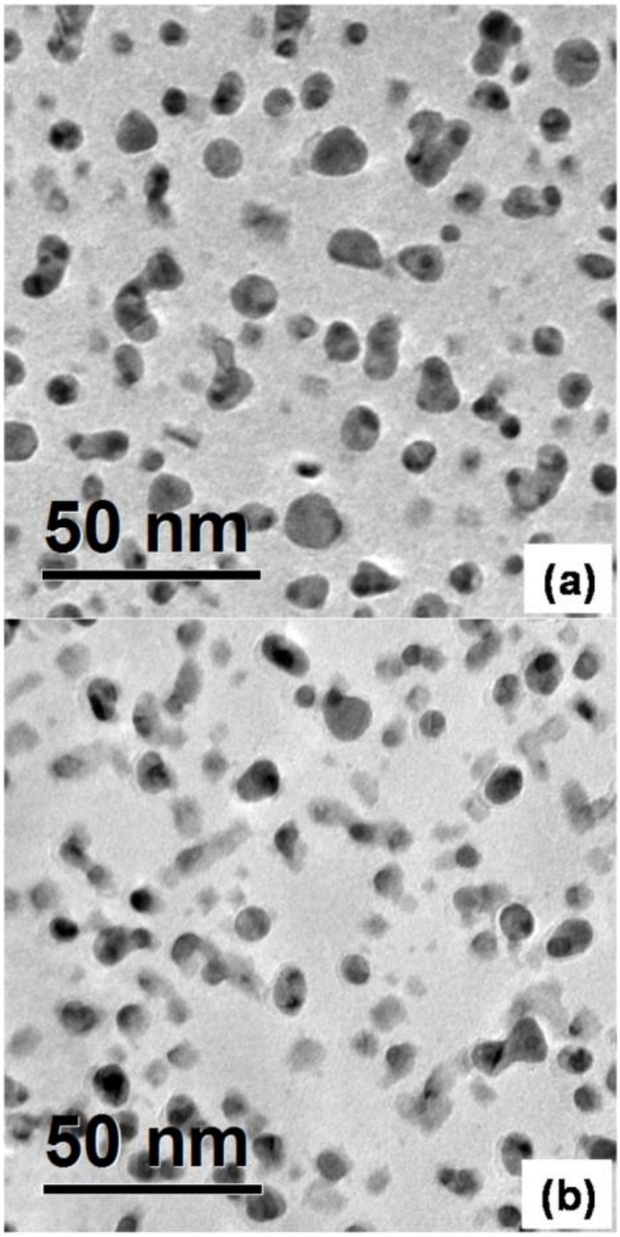

Figure S1: TEM plan view of (a) AuNPs deposition on a bulk substrate, followed by mechanical thinning and gentle milling and (b) AuNPs deposited on the as thinned sample but before ion milling.

Change of the hetero-epitaxial gold (200) peak after HF post deposition treatment of 70 s. We found that an HF post deposition dipping could alter the hetero-epitaxial ratio also in the sample obtained after a plating step of 3 s. The Au signal in this case was very low to be analyzed by XRD but enough to be detected by Selected Area Electron Diffraction. (Figure S2a) is the diffraction pattern of the as-deposited sample while (Figure

S2b) is the SAED acquired under similar conditions of the sample after an HF dip of 70 s. The hetero-epitaxial peak (200) for Gold is very prominent and aligned with the (400) spot of the substrate in the as deposited sample but its intensity decreases after the HF treatment. To be more quantitative the corresponding normalized intensity profiles are reported in (Figure S2c). Also in this case the rearrangement of flat regions is followed by a loss of hetero-epitaxy.

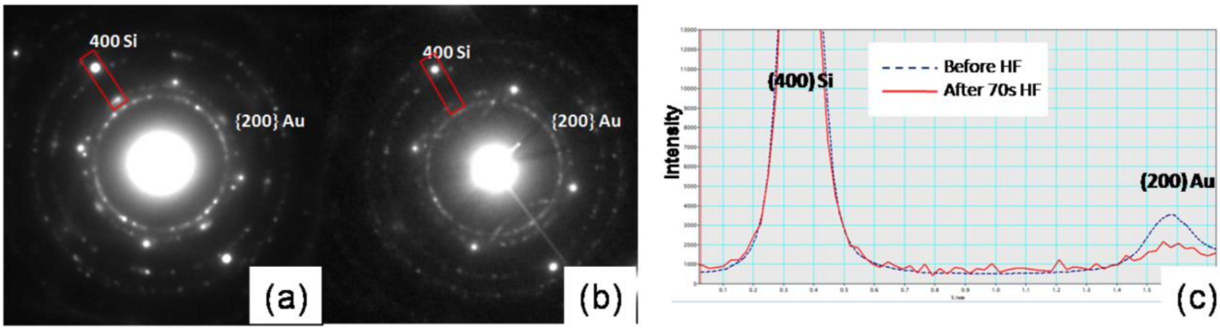

Figure S2: (a) SAED of as deposited sample showing the brighter hetero-epitaxial Au (200) peak and (b) SAED, acquired under quite similar conditions, of the same sample but after HF dip of 70 s. The intensity of the hetero-epitaxial spot is reduced (c).

Atoms rearrange but the gold amount is maintained.

The samples were analyzed by RBS with 2.0 MeV He^+ ions scattered by target atoms at 165° and energy detected by a solid state surface barrier detector of 15 keV resolution. The beam was of about 1 mm^2 size and of few nA intensity. The areal density for Gold was measured before and after the HF treatment and by integration of the corresponding spectra, reported in Figure S3, we found that it doesn't change.

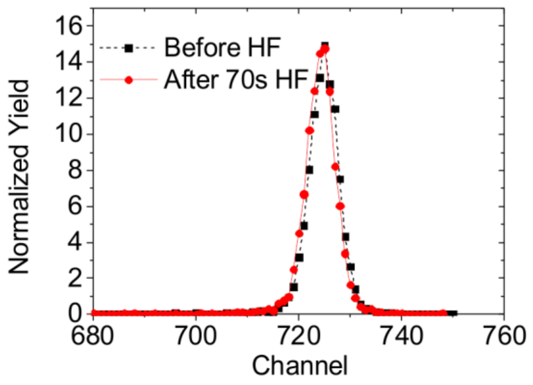

Figure S3: RBS spectra of Au as deposited and after a HF immersion of 70 s.
